# Evaluation of routine care of prurigo nodularis in Germany: retrospective chart review study (ADVANCE PN)

**DOI:** 10.1111/ddg.15721

**Published:** 2025-06-08

**Authors:** Ralph von Kiedrowski, Martin Metz, Elke Weisshaar, Inka Albrecht, Marie Schild, Sonja Ständer

**Affiliations:** ^1^ Company for Medical Study & Service Selters and Private Practice Dermatology Selters/Westerwald Germany; ^2^ Institute of Allergology Charité – Universitätsmedizin Berlin Corporate Member of Freie Universität Berlin and Humboldt‐Universität zu Berlin Berlin Germany; ^3^ Fraunhofer Institute for Translational Medicine and Pharmacology ITMP Immunology and Allergy Berlin Germany; ^4^ Division of Occupational Dermatology Department of Dermatology Ruprecht‐Karls University Heidelberg Heidelberg Germany; ^5^ Sanofi‐Aventis Deutschland GmbH Berlin Germany; ^6^ Center for Chronic Pruritus and Section for Pruritus Medicine of the Department of Dermatology Münster University Hospital Münster Germany

**Keywords:** chronic prurigo, nodules, Prurigo nodularis, real‐world evidence, routine care, treatment pattern

## Abstract

**Background and Objectives:**

Knowledge on patient care gaps of prurigo nodularis (PN) is limited. This retrospective chart review (ADVANCE PN) investigated unmet medical needs and gaps in diagnostics, treatment, and management of patients with PN in routine care in Germany.

**Patients and Methods:**

Medical records for adults newly diagnosed with PN between January 2012 and December 2022 from dermatologic clinics and office‐based dermatologists were analyzed. Baseline demographics, treatment patterns, diagnostics, symptoms, patient‐reported outcomes (PROs), and disease‐specific scores are reported.

**Results:**

Records of 363 patients from 42 sites were analyzed. Median age (range) was 67 (19–95) years; most patients were female (61.7%), Caucasian (73.4%), and retired (57.3%). Overall, 209 (62.2%) patients had comorbidities (most common: hypertension [28.3%]). Clinically, most patients had nodules (81.1%) or papules (66.7%). PROs, disease‐specific scores, and laboratory assessments were performed for 32 (8.8%), 12 (3.3%), and 71 (19.7%) patients, respectively. Topical corticosteroids (TCS) were the most common overall (90.9%) and first‐line therapy (84.9%); for second‐line therapy, ‘no further treatment’ was most commonly documented (58.6%).

**Conclusions:**

The findings of ADVANCE PN indicate a high unmet need in the current state of medical care, evidenced by shortcomings in PRO assessment, PN documentation, and adherence to guidelines on PN.

## INTRODUCTION

Prurigo nodularis (PN) is a chronic neuroimmune skin disease characterized by the presence of chronic pruritus and pruriginous nodules.^1^ PN belongs to the spectrum of chronic prurigo as patients might present with papular, umbilicated, or plaque‐like pruriginous lesions. PN starts with chronic pruritus, resulting in an itch‐scratch cycle leading to the development of the pruriginous lesions. PN is often associated with a diverse array of dermatologic, systemic, neurologic, and/or psychiatric comorbidities, demonstrating the complexity in manifestations of PN.^2^ Patients incur a substantial impairment in quality of life (QoL), which has been reported to be similar to, or worse than, that for other inflammatory skin conditions, such as atopic dermatitis and psoriasis.^3^ The literature reports an estimated prevalence and yearly incidence in Germany of 111 per 100,000 and 20 per 100,000 individuals, respectively.^4^

Treatment of PN remains challenging. Although topical corticosteroids (TCS) have very limited effects when used as treatment for PN, they are most commonly used as a first‐line therapy.^5^ A step‐wise treatment ladder starting with topical treatments and ultraviolet (UV) phototherapy, followed by systemic treatment, is recommended by the German S2K guideline^5^ and by the international guideline for chronic prurigo including PN published in 2021.^6^ After publication of this guideline, the first targeted therapy for PN, dupilumab, was approved in 2022 by the *US Food and Drug Administration*,^7^ and the *European Medicines Agency*.^8^ Data on the use of recommended or approved therapies in PN are still missing as there is no global registry. Based on patient interviews conducted in Europe between 2017 and 2019, it can be concluded that most patients with PN are not satisfied with the therapy they received for the disease.^9^

In addition, a retrospective claims data study of the demographics, disease outcomes, healthcare resource utilization (HCRU), and costs of PN in a real‐world setting in Germany has previously been conducted between 2012 and 2016. The study highlighted the high clinical and economic burden incurred by PN, and showed that further research of PN in Germany is warranted.^10^ Therefore, this retrospective chart review study (ADVANCE PN) was designed to investigate the state of medical care in PN between 2012 and 2022, and to identify gaps in routine care in outpatient dermatological offices. The primary objective was to explore current treatment patterns for PN, using a medical record review‐derived real‐world multicenter cohort of patients diagnosed with PN. The secondary objectives were to examine and describe patient demographic and clinical characteristics, assess patients’ clinical histories, characterize treatment patterns and health outcomes, and evaluate HCRU associated with PN in Germany. With this study, we aim to generate comprehensive data to understand the current state of the art in diagnosis and therapy, and to identify gaps in medical care.

## MATERIAL AND METHODS

### Study design

**FIGURE 1:**
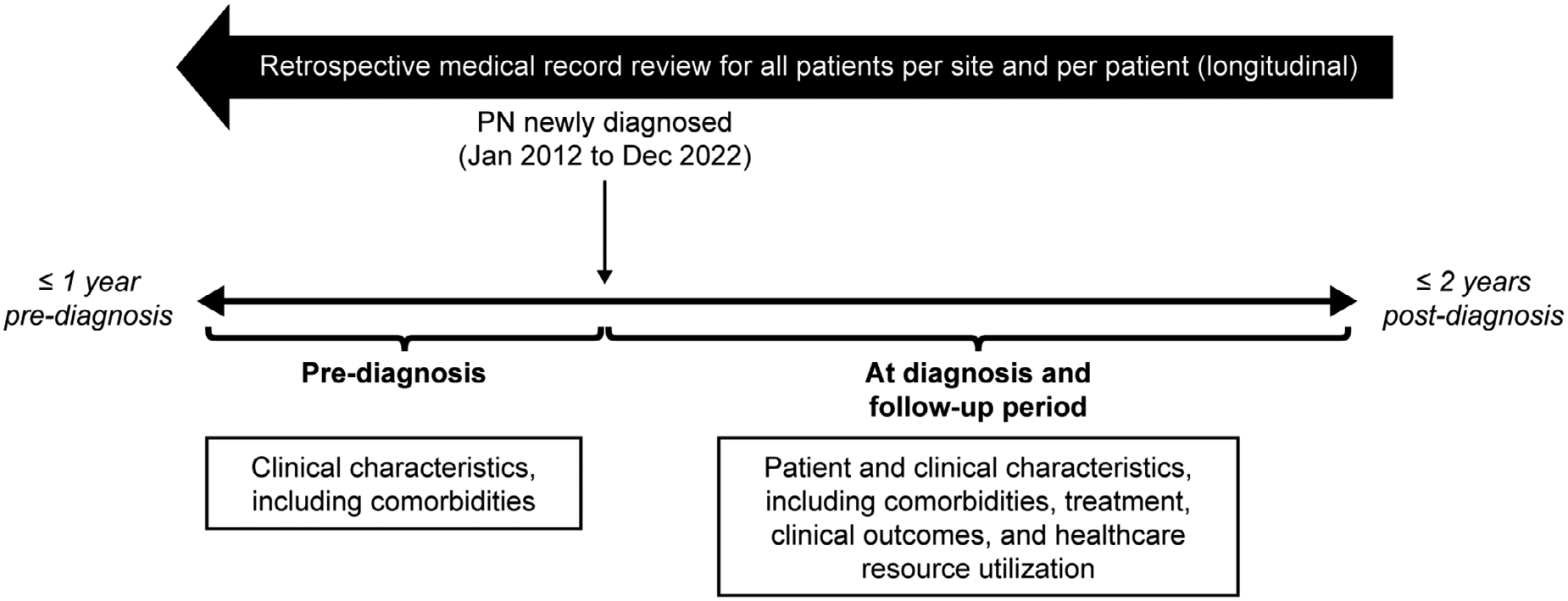
Study design of ADVANCE PN. *Abbr*.: PN, prurigo nodularis

This retrospective review included medical records for adult patients newly diagnosed with PN between January 2012 and December 2022 in Germany. Participating sites were clinic departments of dermatology and dermatologists in private practice which diagnosed and treated ≥ 2 patients with PN in the study time period. Centers specialized on chronic pruritus providing specific pruritus office hours were excluded to minimize site bias and to dissect usual dermatologic care of PN. The target sample size was approximately 250 patients recruited from up to 100 sites. The study design of ADVANCE PN is presented in Figure 1.

### Participants

Adults with a confirmed diagnosis of PN (definite first diagnosis of “prurigo nodularis” or “other prurigo” or “chronic prurigo” made by a dermatologist; diagnoses were coded using International Classification of Diseases, 10th Edition [ICD] 10 codes L28.1, L28.2, or “other”) in the study period were included. Patients were excluded if they were diagnosed with a differential diagnosis of PN (including scabies, prurigo simplex subacuta, dermatitis herpetiformis, hypertrophic lichen planus, lichen amyloidosis, skin‐picking syndrome, and other).

### Data collection

Dermatologists at participating sites entered data from medical records of all patients diagnosed and treated between January 2012 to December 2022. Data were collected retrospectively in electronic case forms at one year before diagnosis, the PN diagnosis visit, and follow up visits ≤ 2 years after diagnosis. The data collection methodology is shown in the online supplement.

### Study size

A target sample size of approximately 250 patients from up to 100 sites was planned for this study. Study size was chosen based on Johnston KM, et al.^11^ For a sample size of 200, and a treatment given to 5% of the population, the precision of a 95% confidence interval (CI) was expected to be ± 0.03 (expected 95% CI, 0.02, 0.08).

### Statistical analysis

The methodology for statistical analyses is shown in the online supplement.

### Ethics approval

This study was approved by an independent ethics committee (Landesärztekammer Rheinland‐Pfalz) and conducted in accordance with the *Declaration of Helsinki*, the *Guidelines for Good Epidemiological Practice*, and all applicable local regulatory requirements.

## Results

### Patient disposition and general site information

**FIGURE 2:**
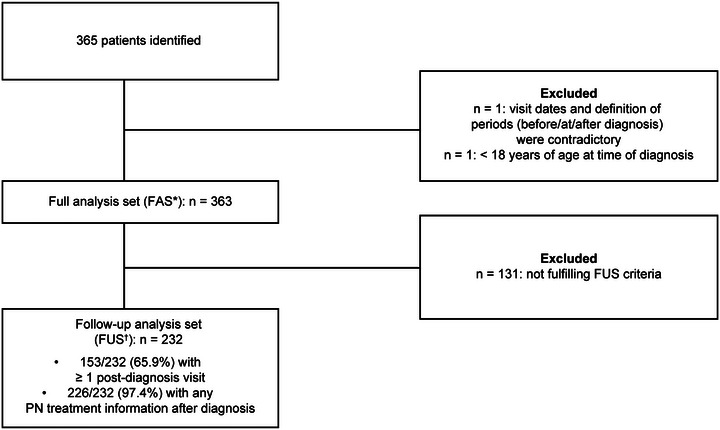
Disposition of patients in ADVANCE PN. *Abbr*.: FUS, follow‐up set; PN, prurigo nodularis *Full Analysis Set (FAS): Adult patients with PN and a documented diagnosis visit and other visits ≤ 1 year before and ≤ 2 years after the diagnosis. ^†^Follow‐up set (FUS): FAS patients with at least ≥ 1 follow‐up visit OR any PN treatment information after diagnosis (first PN treatment with either end date or ongoing, and/or start date of PN treatment after diagnosis).

Overall, records of 365 patients were documented, with 363 (99.5%) included in the full analysis set (FAS; patients with a documented diagnosis visit and other visits ≤1 year before and ≤2 years after the diagnosis). The reasons for exclusion were contradictory information on visit dates and definition of periods “before”/”at”/”after” diagnosis (n = 1) and being aged < 18 years at the time of diagnosis (n = 1). Records of 232 patients (63.6%) were included in the follow‐up set (FUS; FAS patients with at ≥ 1 follow‐up visit or any PN treatment information after diagnosis), with 131 patients excluded for not having ≥1 post‐diagnosis visit or information on any PN treatment after diagnosis (Figure 2).

Patient data were collected from 42 sites. Most sites (n = 39) were private dermatologic practices; three university clinics participated in the study (University Clinic Tuebingen, University Clinic Mainz, Elbe Klinikum, Buxtehude). Of the 30 sites that provided further site information, most (76.7%, n = 23) participated in advanced education and/or training for PN (Table S1, online supplement) and had ≥ 10 patients with PN between the study period dates (83.9%, n = 26); the remainder (16.1%, n = 5) had 2–10 patients with PN. The mean (standard deviation [SD]) number of patients with PN per quarter was 6.5 (5.6).

### Demographics, diagnosis, and disease management

**FIGURE 3:**
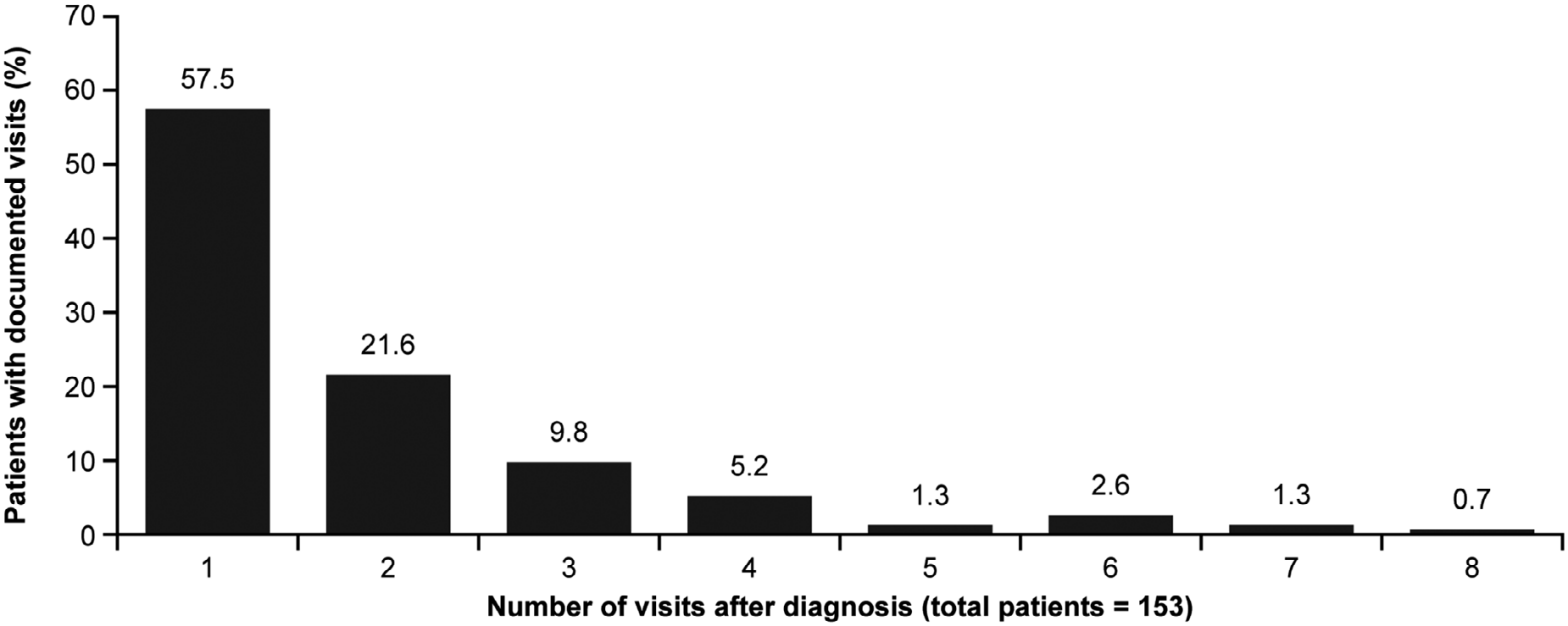
Distribution of the number of post‐diagnosis dermatology clinic visits

From 363 patients in the FAS, 25 patients had a pre‐diagnosis visit (6.9%; clinical details provided in Table S2 in the online supplement), with 23 (92.0%) of these having only one visit and the remainder with two visits. In total, 153 (42.1%) patients had ≥ 1 documented follow‐up visit after diagnosis. The mean (range) number of visits after diagnosis was 1.9 (1–8); the majority of patients (57.5%, 88/153) had only one documented visit after diagnosis, and this proportion decreased with increased number of visits (Figure 3).

Baseline demographics and clinical characteristics (FAS) are presented in Table 1. In summary, the median age of patients was 67 (range: 19–95) years. The majority of patients were female (61.7%), Caucasian (73.4%), and retired (57.3%).

**TABLE 1:** Baseline demographics and clinical characteristics of patients included in ADVANCE PN (FAS).

Characteristic	n = 363
***Sex, n (%)***	
Female/male	224 (61.7)/139 (38.3)
***Age (years)***	
Mean (standard deviation)	63.8 (16.5)
Median (range)	67.0 (19 to 95)
***Employment status/occupation, n (%)*** ^*^	
Employed	77 (34.2)
Sick leave/unable to work	1 (0.4)
Regularly retired	129 (57.3)
Prematurely retired	8 (3.6)
Domestic work	6 (2.7)
Unemployed	3 (1.3)
Engaged in (re‐)education	1 (0.4)
Missing	138
***Race, n (%)*** ^*^	
Caucasian	257 (73.4)
African/African American	1 (0.3)
Asian	5 (1.5)
Other/mixed	87 (24.9)
Missing	13
***Status of patient, n (%)*** ^*^	
Existing patient	170 (49.3)
New patient	175 (50.7)
Missing	18
***Referred by other healthcare professional, n (%)*** ^†^	78 (28.1)
Missing	85
**Any comorbidity before or after diagnosis, n (%)**	209 (62.2)
Missing	27
***Comorbidities present in > 5% of patients, n (%)*** ^*^	
Hypertension	95 (28.3)
Diabetes mellitus type 1/ type 2	61 (18.2)
Endocrine/metabolic dysregulation	58 (17.3)
Atopic dermatitis	50 (14.9)
Mental health/psychosomatic disorder	49 (14.6)
Cardio‐ and cerebrovascular disease	44 (13.1)
Obesity	36 (10.7)
Malignant disease	30 (8.9)
Allergic rhinitis	28 (8.3)
Missing	27

* % values calculated using n values without missing data.

^†^ Patients not referred were deemed to have independently arranged clinic visit; % values calculated using n values without missing data.

In total, 99.7% of patients had signs and symptoms of PN at diagnosis according to Pereira M, et al.^9^ (Table S3, online supplement), with 287 patients (79.1%) diagnosed under ICD10 code L28.1 (prurigo nodularis), 67 (18.5%) with L28.2 (other prurigo), 5 (1.4%) with L30.8 (other dermatitis), 3 (0.8%) with other prurigo eczema, and 1 (0.3%) with L87.1 (reactive perforating collagenosis). Most dermatologists confirmed the diagnosis clinically (84.7%, n* = *287), while two (0.6%) confirmed with histopathology, and 50 (14.7%) performed a combination of both (Table S3, online supplement).

**FIGURE 4:**
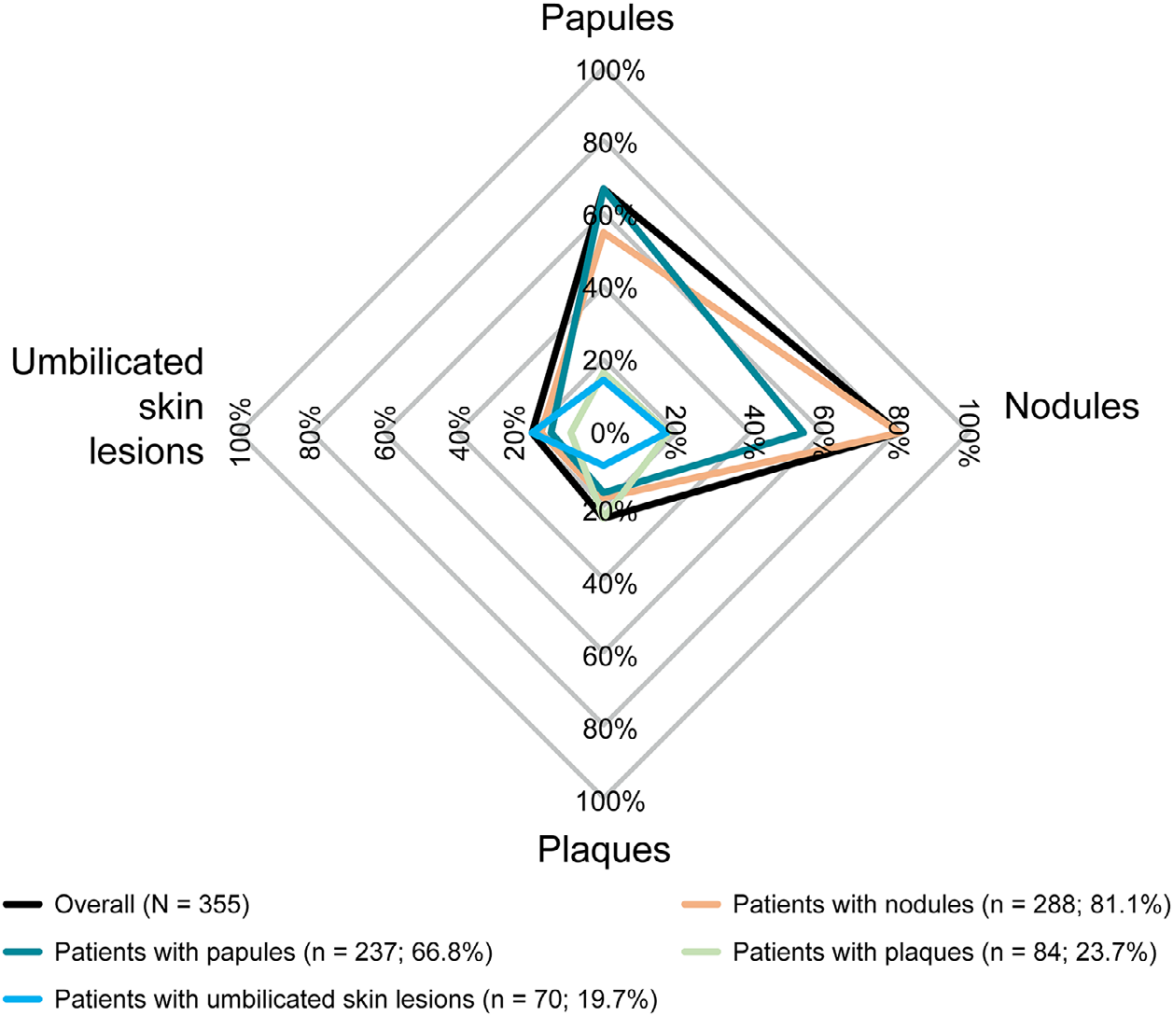
Documented dermatological signs at diagnosis (FAS). Data on dermatological signs at diagnosis in the FAS were not available for eight patients. *Abbr*.: FAS, full analysis set

**FIGURE 5:**
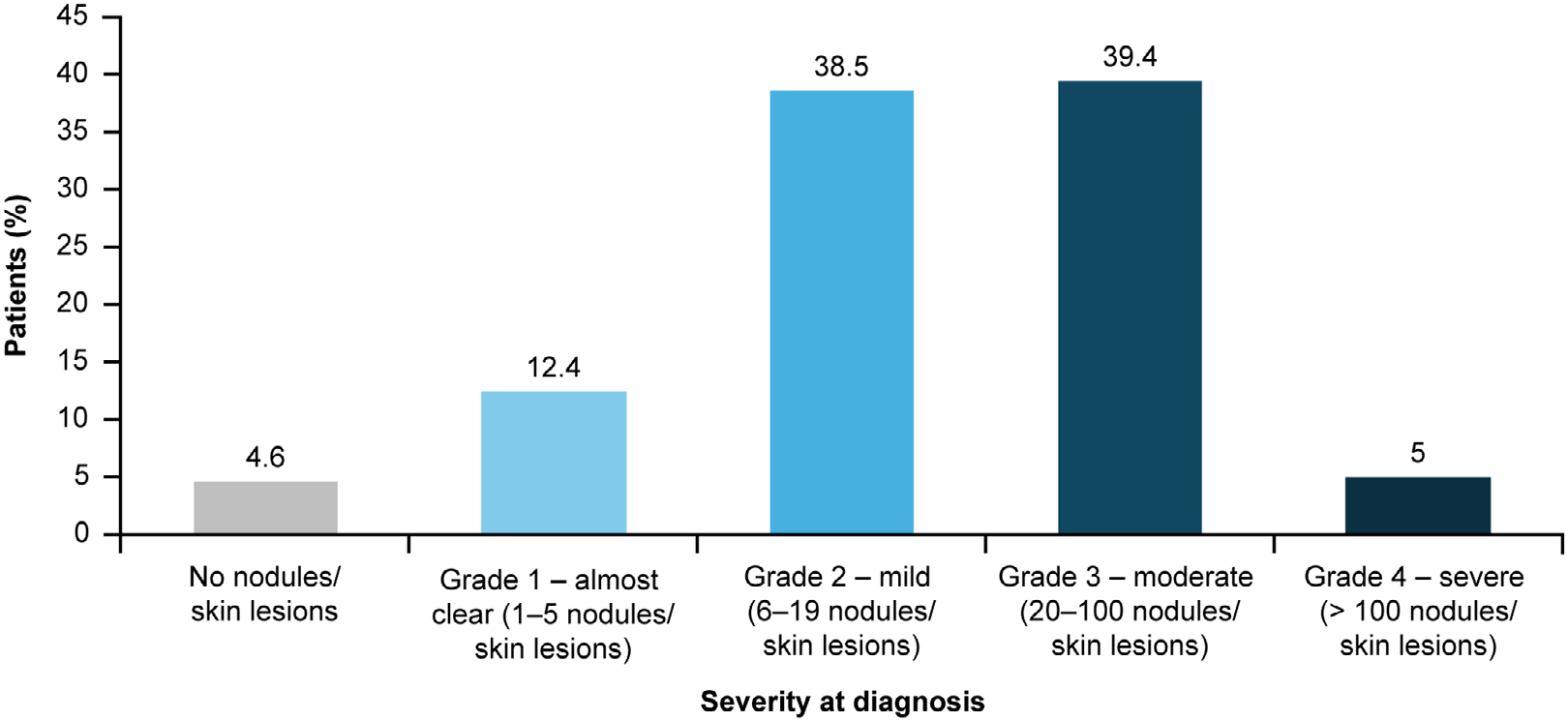
PN severity at diagnosis (FAS). Data reported for 218 patients at diagnosis; no PN severity information was available for 145 patients (40% of the FAS population). *Abbr*.: FAS, full analysis set; PN, prurigo nodularis

**FIGURE 6:**
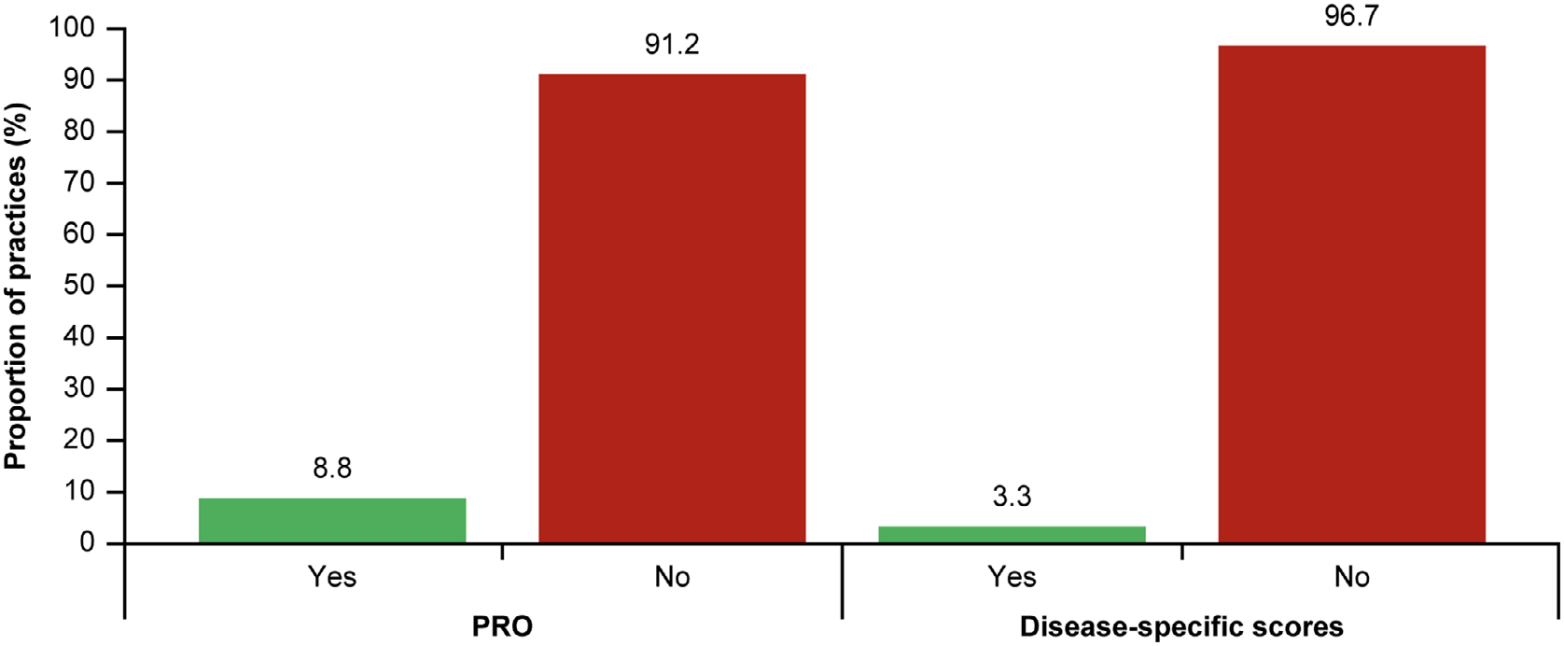
Proportion of practices collecting data for PRO and scores. For n = 34/363 (9.4%) patients, PROs and disease‐specific score assessments were performed and documented. PROs were documented for n = 32 (8.8%) patients, n = 330 (91.2%) had no documented PROs; the PROs used were DLQI (n* = *32/34) and Itch VAS/WI‐NRS (n = 8/34). Disease‐specific scores including IGA‐PN‐S (n* = *2/34) were documented for n = 12 (3.3%) of patients, and n = 350 (96.7%) had no documented scores. Data were missing for n* = *1 patient each for PRO and disease‐specific scores (in total, data for n = 362 patients). *Abbr*.: DLQI, Dermatology Life Quality Index; IGA‐PN‐S, Investigator's Global Assessment for PN‐Stage; PN, prurigo nodularis; PRO, patient reported outcomes; VAS, visual analogue scale; WI‐NRS, Worst Itch Numeric Rating Scale

Most dermatologists documented the following clinical characteristics for diagnosis: chronic pruritus (82.3%, n = 298), “clinical signs or history of repeated scratching” (82.3%, n = 297), and presence of pruriginous lesions (89.2%, n = 321) (Table 2). In total, 288 (81.1%) patients had nodules, 237 (66.8%) had papules, 84 (23.7%) had plaques, and 70 (19.7%) showed umbilicated skin lesions (Figure 4). The most common combination of skin symptoms were nodules and papules (n = 130, 36.6%), followed by nodules, papules and umbilicated pruriginous lesions (n* = *24, 6.8%), and 21 (5.9%) patients reporting all four pruriginous lesions (Table 3). For behavioral and psychological symptoms at diagnosis, 135 (35.0%) patients had PN‐related sleep disturbance documented, and 42 (11.8%) reported depression/anxiety. Of 218 (60.1%) patients with data on PN disease severity at diagnosis, the majority had either mild (approximately 6–19 nodules/skin lesions; n* = *84, 38.5%) or moderate PN disease (approximately 20–100 nodules/skin lesions; n* = *86, n* = *39.4%) (Figure 5); disease severity was rated based on physician estimations (no use of instruments). Only 12 (3.3%) reported use of PN disease‐specific scores such as *Investigator's Global Assessment (IGA)‐PN* (Figure 6).

**TABLE 2:** Dermatological signs and symptoms at diagnosis (FAS).

	Diagnosis n (%)
Chronic pruritus (> 6 weeks)	298 (82.3)
Signs of repeated scratching/scratching during anamnesis	297 (82.3)
Pruriginous lesions	321 (89.2)
Permanent itching skin	184 (51.3)
Sporadic itching skin	165 (46.0)
Burning/stinging skin	79 (22.1)
Skin pain	58 (16.2)
Papules	238 (66.7)
Nodules	292 (81.1)
Plaques	84 (23.5)
Umbilicated skin lesions	70 (19.6)
Ulcerations	43 (12.0)
Hypopigmented maculae	92 (25.8)
Hyperpigmented maculae	105 (29.4)
Relevant sleep disturbance during the night	125 (35.0)
Depression and/or anxiety	42 (11.8)
Other signs and symptoms^*^	9 (2.5)

Information was missing for a small number of patients; however, only valid data have been used for the calculation of percentages.

* Other signs and symptoms: stress‐dependent itch, inner restlessness, excoriation, psychosomatic symptoms, dermatozoa delusion, chronic UV damage with cutis rhomboidalis nuchae, anal eczema, eczema, hypertension, dementia.

**TABLE 3:** Patients with overlapping PN‐type skin manifestations at diagnosis (FAS).

PN‐type symptom				n	%
Papules	Nodules	Plaques	Umbilicated skin lesions		
X	X	–	–	130	36.6
–	X	–	–	61	17.2
X	–	–	–	24	6.8
X	X	–	X	24	6.8
X	X	X	X	21	5.9
X	X	X	–	19	5.4
–	–	–	–	18	5.1
–	X	X	–	17	4.8
X	–	X	–	13	3.7
–	X	–	X	10	2.8
–	X	X	X	6	1.7
X	–	X	X	5	1.4
–	–	–	X	3	0.8
–	–	X	–	3	0.8
X	–	–	X	1	0.30
			Total^*^:	355	100.00

* For eight patients, data on symptoms were incomplete and could not be included in this analysis.

*Abbr*.: –, Symptom not present; PN, prurigo nodularis; X, Symptom present

Dermatology practices collected patient‐reported outcome (PRO) data for only 32 (8.8%) of patients (Figure 6), including *Dermatology Life Quality Index* (DLQI) and pruritus intensity via *Visual Analog Scale* (VAS) or *Worst‐Itch‐Numerical Rating Scal*e (WI‐NRS). In particular, itch measurements by VAS or WI‐NRS (PRO) were performed for only eight (2.2%) of these patients.

Of 363 patients in the FAS, 71 (19.7%) had data for laboratory assessments (Table S4, online supplement). The most common were hematology (n = 57, 80.3%), differential blood count (n = 59, 83.1%), liver function (n = 61, 85.9%), kidney function (n = 59, 83.1%), C‐reactive protein (n = 45, 63.4%), and total immunoglobulin (Ig) E (n = 34, 47.9%). In total, 30 patients were hospitalized, and ten visited a specialist before diagnosis (Table 4). The mean (range) duration of time spent in hospital was 11.6 (3–39) days. The most common specialists who patients were referred to were dermatological centers (n = 6), followed by psychologic centers (n = 1), internal medicine (n = 1), and general practitioner (n = 1).

**TABLE 4:** Hospitalizations, healthcare visits, and invalidity due to PN (FAS).

**Healthcare resource**	
***In‐patient hospital stay, n (%)***	
Yes	30 (8.3)
No/not documented	330 (91.7)
Missing	3
Total	360 (100.0)
***Emergency room visits, n (%)***	
Yes	1 (0.3)
No/not documented	360 (99.7)
Missing	2
Total	361 (100.0)
***Specialist/GP visit*** ^*^ ***, n (%)***	
Yes	10 (2.8)
Dermatologist/psychologist/internal medicine clinic/GP	6/2/1/1
No/not documented	349 (97.2)
Missing	4
Total	359 (100.0)
***Invalidity/sick leave, n (%)***	
Yes	7 (1.9)
No/not documented	356 (98.1)
Missing	0
Total	363 (100.0)
***Permanent invalidity, n (%)***	
Yes	2 (0.6)
No/not documented	360 (99.4)
Missing	1
Total	362 (100.0)

*Abbr*.: FAS, full analysis set; GP, general practitioner

### Treatment and treatment patterns

Of the 232 patients in the FUS, 227 (97.8%) received treatment for PN (Table 5). TCS were the most common (90.9%, n* = *211) treatment, followed by antihistamines (28.4%, n = 66), and UV‐therapy (22.0%, n = 51). Other therapies had been received by 21.6% (n = 50) including biologics in ten of these patients.

**TABLE 5:** Treatments in the FUS population at any time throughout the study period.

Treatment at any time, n (%)	n = 232
Any	227 (97.8)
TCS	211 (90.9)
Antihistamines	66 (28.4)
UV	51 (22.0)
Other therapies including biologics^*^	50 (21.6)
Systemic corticosteroids	16 (6.9)
Antidepressants	11 (4.7)
TCI	11 (4.7)
Gabapentinoids	10 (4.3)
Capsaicin	7 (3.0)
IMP of clinical study	6 (2.6)
Immunosuppressives	5 (2.2)
Opioid‐R‐antagonists	3 (1.3)
Neurokinin 1 receptor (NK1R) antagonists	2 (0.9)

* Among ‘other therapies’, n = 10 (4.3%) received biologics.

*Abbr*.: IMP, investigational medicinal product; TCI, topical calcineurin inhibitors; TCS, topical corticosteroids; UV, ultraviolet phototherapy

**FIGURE 7:**
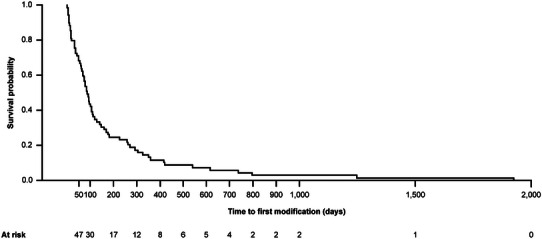
Kaplan‐Meier analysis of time to first treatment modification (FUS; with data imputation). Kaplan‐Meier analysis of time to first treatment modification (days), n* = *179: Mean (standard deviation): 108.9 (199.0) days. Median (interquartile range): 90.0 (25.0 to 90.0) days. *Abbr*.: FUS, follow‐up set

**FIGURE 8:**
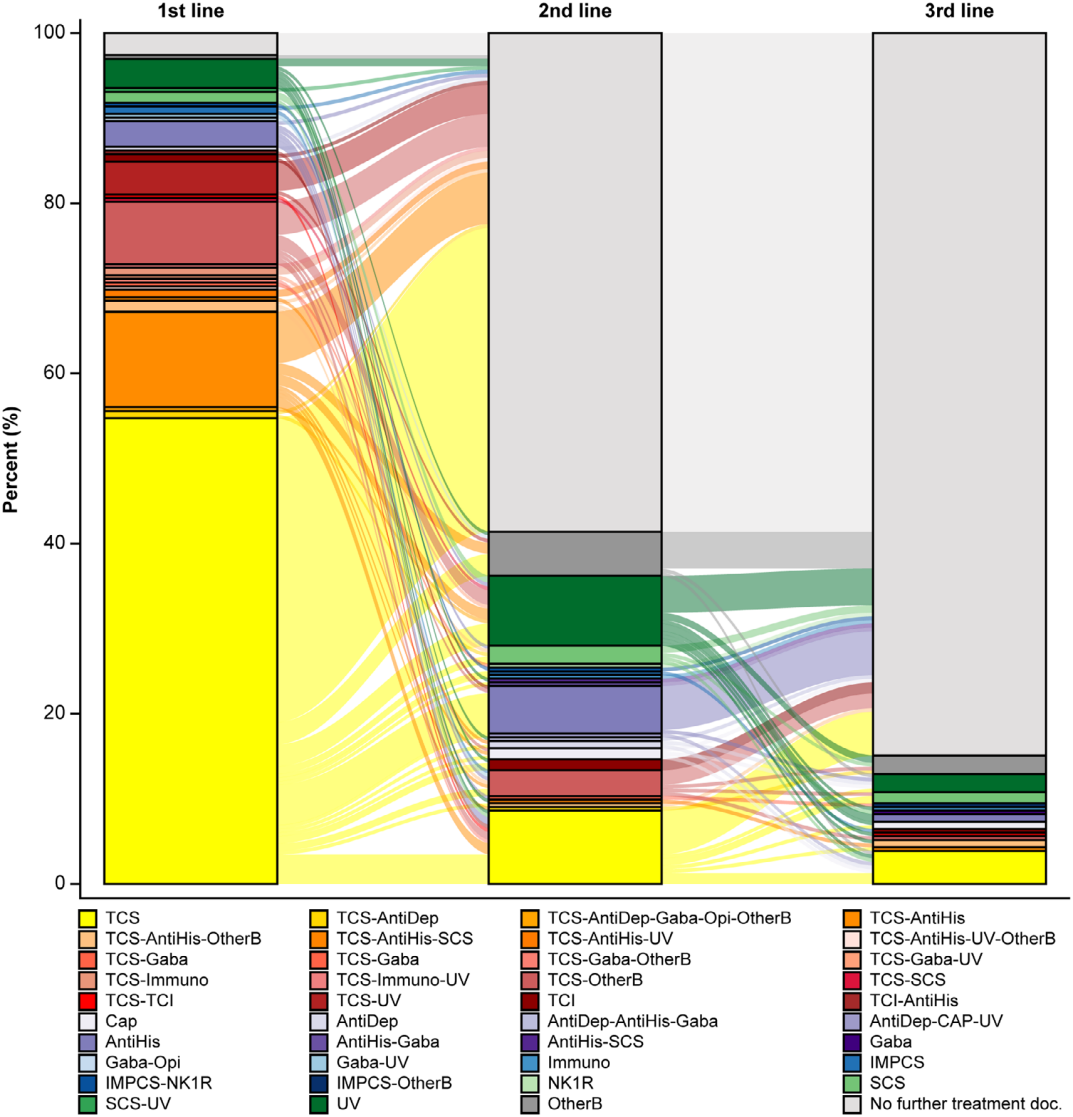
Treatment patterns across lines of therapy (Sankey diagram). *Abbr*.: AntiDep, antidepressants; AntiHis, antihistamines; Cap, capsaicin; FUS, follow‐up set; Gaba, gabapentinoids; IMPCS, investigational medicinal product of clinical study; NK1R, Neurokinin 1 receptor antagonists; Opi, Opioid‐R‐antagonists; OtherB, other treatments including biologics; PN, prurigo nodularis; SCS, systemic corticosteroids; TCI, topical calcineurin inhibitors; TCS, topical corticosteroids; UV, ultra‐violet phototherapy

The median time until first treatment modification (with data imputation) was 90 (range: 0 to 1924) days (n* = *179; Figure 7). The results of the sensitivity analysis on data without imputation showed similar findings (median 86 days; range 0–1924; n* = *69). The treatment types by line of therapy are shown in Table 6 (FUS, n* = *232). Treatment with TCS was the most commonly used treatment for first‐line (84.9%, n* = *197), second‐line (13.4%, n* = *31), and third‐line therapy (6.0%, n* = *14). The treatment patterns of patients with PN are presented in Figure 8. As first‐line therapy, most patients (85%, 197/232) received TCS, either as monotherapy (54.7%, 127/232) or as combination therapy (30.2%, 70/232). For six patients (2.6%) no treatment was documented. Additionally, 58.6% of patients had no documented second‐line treatment. TCS monotherapy was second‐line treatment for 8.6%. For third‐line therapy, “no further treatment documented” was reported for 84.9% of patients. 3.9% received TCS monotherapy as third‐line treatment (Figure 8).

**TABLE 6:** Treatment types by line of therapy (FUS).

	First line		Second line	Third line
	*All (n = 232)*	*Excluding ongoing treatment at diagnosis*		
No treatment documented, n (%)	6 (2.6)	21 (9.1)^‡^	136 (58.6)^*^	197 (84.9)*
Any, n (%)	226 (97.4)	211 (90.9)	96 (41.4)	35 (15.1)
TCS	197 (84.9)*	184 (79.3)*	31 (13.4)^†^	14 (6.0)^†^
Antihistamines	42 (18.1)^†^	38 (16.4)^†^	18 (7.8)^‡^	5 (2.2)^‡^
Other therapies including biologics	25 (10.8)^‡^	21 (9.1)^‡^	20 (8.6)^‡^	9 (3.9)^‡^
UV	24 (10.3)^‡^	23 (9.9)^‡^	21 (9.1)^‡^	5 (2.2)^‡^
Systemic corticosteroids	6 (2.6)	5 (2.2)	6 (2.6)	3 (1.3)
Antidepressants	5 (2.2)	3 (1.3)	5 (2.2)	–
Gabapentinoids	5 (2.2)	3 (1.3)	3 (1.3)	1 (0.4)
TCI	4 (1.7)	4 (1.7)	3 (1.3)	2 (0.9)
IMP of clinical study	3 (1.3)	3 (1.3)	2 (0.9)	1 (0.4)
Immunosuppressives	3 (1.3)	3 (1.3)	1 (0.4)	1 (0.4)
Opioid‐R‐antagonists	2 (0.9)	–	–	–
Capsaicin	1 (0.4)	1 (0.4)	4 (1.7)	2 (0.9)
Neurokinin 1 receptor (NK1R antagonists	1 (0.4)	1 (0.4)	2 (0.9)	–

* Most common therapy at each time point.

^†^ Second most common therapy.

^‡^ Third most common.

*Abbr*.: IMP, investigational medicinal product; TCI, topical calcineurin inhibitors; TCS, topical corticosteroids; UV, ultraviolet phototherapy

## Discussion

ADVANCE PN was a retrospective review of medical records for 363 adults newly diagnosed with PN between January 2012 and December 2022 in real‐world settings in mostly private dermatological practices in Germany. Most patients had mild to moderate clinical disease activity comprising all pruriginous lesion types. Only 66.3% of patients had data for a follow‐up visit or any treatment information after diagnosis. There was a marked decrease in the number of dermatological site visits after diagnosis, with most patients only attending one follow‐up, and less than one‐quarter of patients attending two follow‐up visits. This decrease could be partially due to patients switching to different physicians, and we were unable to separate those patients from those who terminated treatment. Other reasons include a strong dissatisfaction with available therapies, as shown in earlier studies.^9^

In terms of diagnostics, variation between the ICD10 code used by dermatologists occurred, and most diagnoses were performed clinically and on site without histopathologic confirmation, highlighting a lack of uniformity. This observation, in addition to the sparse number of follow‐up visits, indicates a need for improved care of patients with PN post‐diagnosis. Furthermore, 67 (18.9%) patients in ADVANCE PN presented with PN and no nodules, highlighting the variation in physical manifestations of the disease. The inconsistent use of ICD10 diagnostic codes, and the observed proportion of patients without nodules, support the proposed shift in terminology used to describe this disease from PN to chronic prurigo,^12^ and in examples of patients with nodules, the use of the term “chronic nodular prurigo” used as a subtype of chronic prurigo.^13^

For baseline characteristics, a similar proportion of females with PN were reported in ADVANCE PN (61.7%) compared with studies of a German center specializing in pruritus conducted between October 2004 and February 2018, including a retrospective analysis of 1,128 patients (62.2% female) with chronic prurigo.^14^ Patients in ADVANCE PN were slightly older (median age 67 years) and mostly retired compared with those in the pruritus specialist center study (median age 63–64 years).^14^, ^15^, ^16^ Furthermore, hypertension (28.3%) and diabetes mellitus (18.2%) were the most reported comorbidities. The proportion of patients with diabetes was consistent with published data of pruritus centers (18.4–22.7%).^10^, ^17^ In contrast, the proportion of patients with hypertension and atopic diathesis was higher in analyses of pruritus centers (hypertension: 44.3–51.1%;^10^, ^17^ atopic diathesis: 42.2%,^16^, ^17^ respectively), compared with our study (atopic dermatitis: 14.9%) and results of a retrospective, cross‐sectional claims data analysis (13.6%).^10^ The proportion of patients with mental disease was lower in ADVANCE PN (14.6%) compared with other studies from pruritus centers (24.3%),^17^ indicating a higher sensibility in documenting these manifestations in pruritus centers versus routine care in Germany.^14^ In general, the observed differences might be attributable to variations in study design and the characteristics of participating sites, reflecting the management of distinct patient populations in general care versus specialized centers with focus on chronic pruritus.

PN is associated with a high itch intensity, high disease burden and poor quality of life. As treatment targets exist, the *International Forum for the Study of Itch Guidelines* recommend the use of itch intensity scales (such as NRS) and PROs (including DLQI) to assess the severity and burden of PN in patients.^6^ Only 32 (8.8%) and 12 (3.3%) patients had PRO and disease‐specific scores assessments, respectively, in ADVANCE PN. Furthermore, the guidelines recommend the collection of several laboratory parameters, in order to identify possible etiological factors underlying the disease, and therefore assist the tailoring of treatment decisions to individual patients;^6^ however, only 71 (19.6%) patients in ADVANCE PN had laboratory assessments. This highlights a lack of adherence to the guidelines, even with assessments that are simple for dermatologists to conduct and of high relevance for disease management, including measurement of itch intensity. This is key in the management of the disease as patients suffer longer and with higher intensity from pruritus.^16^

As until recently there was no therapy approved for PN, the guidelines recommend a multi‐modal approach aiming to reduce itch and the number and size of skin lesions. This approach includes a “treatment ladder” to escalate treatment as required to target the symptoms of PN through various pathways, involving a stepwise escalation of potential therapies.^5^ TCS were the most common treatment reported in ADVANCE PN (90.9%); however, other studies have shown gabapentinoids and immunosuppressants to be the most successful therapeutic agents. In these previous studies, it was demonstrated that patients with PN require a prolonged duration of therapy;^15^ however, in ADVANCE PN, there was a marked increase observed in no treatment being documented as the second and third line therapy for the majority of patients. This might indicate an underestimation of treatment required for the management of PN as shown by other studies.^6^ Of note, a study of 131 adults with chronic nodular prurigo reported a high dissatisfaction of patients regarding therapy; the most commonly used were emollients, TCS, and antihistamines, and no patients received potent systemic therapy.^13^ Receiving a clear diagnosis and treatment was documented as “very important” as reported by 93.6% of patients in a retrospective explorative study of 1,711 patients with chronic prurigo.^16^ The findings of ADVANCE PN highlight the lack of uniformity and clarity in diagnostics and treatment in routine care; this indicates an unmet need in the management of patients with chronic prurigo, evidenced by the lack of adherence to treatment ladder recommended in the guidelines.^5^

### Strengths and limitations

The chart review design employed in ADVANCE PN enabled collection of laboratory assessments performed, PROs, and disease‐specific scores, all of which are often limited in real‐world studies of patients with PN. Also, the small number of pruritus centers in Germany were excluded from ADVANCE PN, as patients in these centers received off‐label therapies, in accordance with guideline recommendations for the management of these patients; this might not provide an accurate representation of treatment strategies of PN in dermatological practice in a broader context. The limitations of ADVANCE PN include that the HCRU assessment did not include the consideration of treatment use, as this is often managed by further disciplines, such as general practitioners. Further limitations of this study are its retrospective design, the characteristics of the German healthcare system (for example, the allowance to prescribe certain topical and systemic treatments against itch), the lack of uniformity in the management of PN by general practitioners or for inpatients, and a variable expertise in chronic prurigo/PN in ambulatory dermatological care. These factors may have influenced the completeness of data recorded in the questionnaire. Moreover, although we have robust data for the treatments that patients first received, the lack of follow‐up means that we do not have true representativeness of the durability in responses to treatment, and as previously mentioned, patients who switched to different physicians and continued their treatment could appear in our results as a treatment discontinuation, and therefore an overestimation of “no further treatment” would exist. Furthermore, the sample size of patients with data for symptoms before diagnosis was too small to deduce any conclusions, and patients with no end date might have discontinued or switched their treatment to other physicians, and by imputing the data, treatment duration might be overestimated.

## CONCLUSIONS

Our findings highlight a lack of adherence to the guideline recommendations in terms of the assessments performed by dermatologists and treatment algorithms in PN. The low usage of PN‐specific disease measures and scores might indicate an underestimation of disease severity and burden. Future analysis might reveal differences to disease management in expert pruritus centers and advances in disease management after approval of targeted systemic treatments including dupilumab.

## FUNDING

Sponsorship for this study was funded by Sanofi and Regeneron.

## CONFLICT OF INTEREST STATEMENT

R.K. has provided consultancy and served on advisory boards and as a speaker for AbbVie, ALK Scherax, Almirall Hermal, Amgen, Beiersdorf Dermo Medical, Biofrontera, Biogen, BMS, Boehringer Ingelheim, Celgene, Celltrion HC, DermaPharm, Foamix, Galderma, Gilead, Hexal, Incyte, Janssen‐Cilag, LEO Pharma, Lilly Pharma, Medac, Menlo, MSD, Mylan/Viatris, Novartis, Dr. R. Pfleger, Pfizer, Regeneron, Sanofi, Stada, Stallergens, Stiefel GSK, Tigercut, and UCB. M.M. has provided consultancy and served on advisory boards and as a speaker for AbbVie, ALK‐Abello, Almirall, Amgen, AstraZeneca, Argenx, Bayer, Celgene, Celldex, Celltrion, Escient, Galderma, Grünenthal, GSK, Incyte, Jasper, Menlo, Moxie, Novartis, Pharvaris, Pfizer, Regeneron, Roche, Sanofi, Teva, ThirdHarmonicBio, and Vifor. E.W. has served on advisory boards for Menlo and Sanofi. They also regularly advise and treat patients with chronic itch and chronic prurigo; they are first author of “European Guideline on Chronic Pruritus”, 2023, and last author of the S2k‐guideline “Diagnostik und Therapie des chronischen Pruritus”, AWMF‐Register‐Nr.: 013–048, 2021. They are also co‐author of “IFSI‐guideline on chronic prurigo including prurigo nodularis“, 2020, and founder and current president of the International Forum for the Study of Itch (IFSI). I.A. and M.S. are employees of Sanofi, and may hold stock and/or stock options in the company. R.H. is an employee of GKM Gesellschaft für Therapieforschung mbH who conducted the statistical analysis sponsored by Sanofi. S.S. has provided consultancy for AbbVie, Almirall, Beiersdorf, Clexio, Escient, Galderma, Grünenthal, Incyte, IntegrityCE, Kiniksa, Klinge Pharma, Lilly, P.G. Unna Academy, Pfizer, Sanofi, TouchIME, Vifor, and WebMD. They have also served on advisory boards for AbbVie, Almirall, Galderma, Lilly, Pfizer, Sanofi, and Vifor, and served as a speaker for AbbVie, BMS, FomF, Galderma, LeoPharma, L`Oreal, MEDahead, Moroscience, Novartis, Sanofi, P. G. Unna Academy, Pfizer, UCB, and Vifor.

## References

[1] Zeidler C , Tsianakas A , Pereira M , et al. Chronic prurigo of nodular type: A review. Acta Derm Venereol. 2018;98:173‐179. 29135018 10.2340/00015555-2774

[2] Williams KA , Huang AH , Belzberg M , et al. Prurigo nodularis: Pathogenesis and management. J Am Acad Dermatol. 2020;83:1567‐1575. 32461078 10.1016/j.jaad.2020.04.182

[3] Steinke S , Zeidler C , Riepe C , et al. Humanistic burden of chronic pruritus in patients with inflammatory dermatoses: Results of the european academy of dermatology and venereology network on assessment of severity and burden of pruritus (prunet) cross‐sectional trial. J Am Acad Dermatol. 2018;79:457‐463. e455. 30119869 10.1016/j.jaad.2018.04.044

[4] Ständer S , Ketz M , Kossack N , et al. Epidemiology of prurigo nodularis compared with psoriasis in germany: A claims database analysis. Acta Derm Venereol. 2020;100:adv00309. 33021323 10.2340/00015555-3655 PMC9309863

[5] Ständer S , Zeidler C , Augustin M , et al. S2k guideline: Diagnosis and treatment of chronic pruritus. J Dtsch Dermatol Ges. 2022;20:1387‐1402. 36252071 10.1111/ddg.14830

[6] Ständer S , Pereira MP , Berger T , et al. Ifsi‐guideline on chronic prurigo including prurigo nodularis. Itch. 2020;5:e42.

[7] US Food and Drug Administration . Dupixent® (dupilumab) injection for subcutaneous use. 2017. Available from: https://www.accessdata.fda.gov/drugsatfda_docs/label/2017/761055lbl.pdf [Last accessed June 10, 2024].

[8] European Medicines Agency . Dupixent® (dupilumab) summary of product characteristics. 2017. Available from: https://www.ema.europa.eu/en/medicines/human/EPAR/dupixent [Last accessed June 10, 2024].

[9] Pereira MP , Zeidler C , Wallengren J , et al. Chronic nodular prurigo: A european cross‐sectional study of patient perspectives on therapeutic goals and satisfaction. Acta Derm Venereol. 2021;101:adv00403. 33320272 10.2340/00015555-3726 PMC9366694

[10] Ständer S , Ketz M , Akumo D , et al. Comorbidities, healthcare resource utilization & treatment pattern among patients with prurigo nodularis, compared to a benchmark in germany: A real‐world evidence claims data study. J Eur Acad Dermatol Venereol. 2024;38:883‐894. 38078642 10.1111/jdv.19700

[11] Johnston KM , Lakzadeh P , Donato BMK , et al. Methods of sample size calculation in descriptive retrospective burden of illness studies. BMC Med Res Methodol. 2019;19:9. 30626343 10.1186/s12874-018-0657-9 PMC6325730

[12] Pereira MP , Steinke S , Zeidler C , et al. European academy of dermatology and venereology european prurigo project: Expert consensus on the definition, classification and terminology of chronic prurigo. J Eur Acad Dermatol Venereol. 2018;32:1059‐1065. 28857299 10.1111/jdv.14570

[13] Zeidler C , Pereira MP , Storck M , et al. Severity stages of chronic nodular prurigo: Analysis of associated itch intensity and quality of life impairment. Itch. 2022;7:e61.

[14] Zeidler C , Pereira MP , Ständer S . Chronic prurigo: Similar clinical profile and burden across clinical phenotypes. Front Med (Lausanne). 2021;8:649332. 34268319 10.3389/fmed.2021.649332 PMC8277241

[15] Gründel S , Pereira MP , Storck M , et al. Analysis of 325 patients with chronic nodular prurigo: Clinics, burden of disease and course of treatment. Acta Derm Venereol. 2020;100:adv00269. 32556359 10.2340/00015555-3571 PMC9234994

[16] Zeidler C , Pereira MP , Dugas M , et al. The burden in chronic prurigo: Patients with chronic prurigo suffer more than patients with chronic pruritus on non‐lesional skin. J Eur Acad Dermatol Venereol. 2021;35:738‐743. 32924186 10.1111/jdv.16929

[17] Pereira MP , Hoffmann V , Weisshaar E , et al. Chronic nodular prurigo: Clinical profile and burden. A european cross‐sectional study. J Eur Acad Dermatol Venereol. 2020;34:2373‐2383. 32078192 10.1111/jdv.16309

